# Cannabis April 20th Celebration and Related Emergency Department Visits

**DOI:** 10.1001/jamanetworkopen.2025.11635

**Published:** 2025-05-21

**Authors:** Kevin Lin, Anupam B. Jena, Rosalie L. Pacula, Haiden A. Huskamp, Ateev Mehrotra

**Affiliations:** 1 Department of Health Care Policy, Harvard Medical School, Boston, Massachusetts; 2 Department of Health Policy and Management, University of Southern California (USC) Sol Price School of Public Policy and USC Schaeffer Center for Health Policy and Economics, Los Angeles, California; 3 Department of Health Services, Policy and Practice, Brown School of Public Health, Providence, Rhode Island

## Abstract

This cross-sectional study examines the association between April 20th cannabis celebrations and rates of cannabis-related emergency department visits in the US.

## Introduction

Increasing cannabis use in the US has been associated with cannabis-related adverse events, including cannabis-related emergency department (ED) visits, particularly among young adults.^1^ There has been limited research on what circumstances may drive these visits.

April 20th marks an annual celebration of the counterculture holiday “420,” when typically young adults use cannabis often in large social gatherings. We used this holiday to consider whether potential social drivers of cannabis use (marketing, price promotions, and peer use encouraged by the holiday) are associated with increased ED visits.^2^ We examined rates of cannabis-related ED visits, including among patient subgroups and types of ED visits, on April 20th to understand whether more adverse health events occur on this day.

## Methods

This cross-sectional study used deidentified US national administrative claims data from the Optum Labs Data Warehouse,^3^ including commercial insurance enrollees aged 64 years or younger. We identified all patients with a cannabis-related ED visit on each day of 2016 through 2023 (eMethods in Supplement 1). Race and ethnicity were not accurately captured in these data and therefore not analyzed.

We calculated the risk ratio (RR) of the total count of cannabis-related ED visits on April 20th to the mean number of visits on the days 1 week before and after (April 13th, April 27th) and across various subgroups, including differing levels of state legal medical and recreational dispensary access.^4^ To explore whether the rate of ED visits differed across co-occurring diagnoses (mental illness, gastrointestinal, and other), we used additional non–cannabis-related diagnoses (eMethods in Supplement 1).

We performed a falsification analysis by calculating the same ratios for March 20th (vs March 13th and 27th) and May 20th (vs May 13th and 27th). Recognizing that the COVID-19 pandemic may have limited large social groups, we conducted a sensitivity analysis excluding 2020 data.

Harvard Medical School’s institutional review board waived the informed consent requirement given we used deidentified administrative records. We followed the STROBE reporting guideline.

## Results

In 2023, there were 23 812 796 individuals in our sample (49.8% female; mean [SD] age, 33.6 [17.6] years). Across the full study period, there were 663 cannabis-related ED visits on April 20th and a mean of 568.5 cannabis-related ED visits across control dates (RR, 1.17; 95% CI, 1.04-1.30) (Figure). In subgroup analyses, this association was largest for younger adults (eg, RR for 25-34 years, 1.30; 95% CI, 1.04-1.62) compared with other age groups and largely driven by mental health symptoms such as psychosis (RR, 1.23; 95% CI, 1.03-1.47). The association between April 20th and increased ED visits was not larger in states with legal medical or recreational dispensaries.

**Figure.**
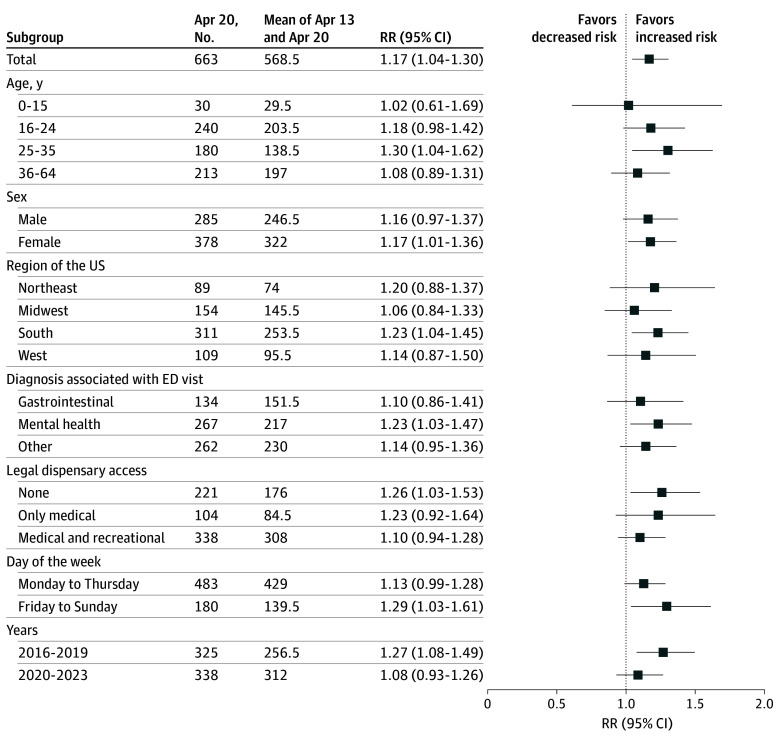
Association Between Cannabis-Related ED Visits and April 20th vs the Mean Number of Visits on Control Days (April 13th and April 27th) Overall and by Subgroup, 2016-2023 RR indicates risk ratio.

Falsification tests showed no substantial difference between March 20th (RR, 1.00; 95% CI, 0.89-1.12) and May 20th (RR, 1.01; 95% CI, 0.90-1.13) and their respective control days. The association was similar after excluding 2020 data (RR, 1.19; 95% CI, 1.05-1.33).

## Discussion

In the first national study on this topic to our knowledge, cannabis-related ED visits on April 20th were 17% higher than on control days. Our findings are consistent in both direction and magnitude with prior work examining the association between April 20th and ED visits in a small sample of British Columbian hospitals and between April 20th and fatal motor vehicle accidents in the US.^2,5^

Future research should explore which social mechanism is driving the excess cannabis use during this holiday (eg, peer-facilitated social pressures or excessive cannabis promotion, marketing, and price discounts). If accidental consumption is responsible, policy recommendations might include broad bans on promotions of high-potency products or regulating high-volume sales.

Our analysis has limitations. First, the number of ED visits on a given day was modest, and therefore our estimates for subgroups are imprecise with large CIs. Second, we defined cannabis-related ED visits based on administrative diagnosis codes, which may not capture all instances in which cannabis use prompted an ED visit.

## Notes

Supplement 1.
eMethods.

Supplement 2.
Data Sharing Statement
