# Supplementary material for: Cannabis April 20th Celebration and Related Emergency Department Visits
Source: JAMA Netw Open. 2025 May 21;8(5):e2511635. doi: 10.1001/jamanetworkopen.2025.11635 (PMC12096254; doi:10.1001/jamanetworkopen.2025.11635)
Supplement: Supplement 1. — eMethods. [file jamanetwopen-e2511635-s001.pdf]

Supplemental Online Content

Lin K, Jena AB, Pacula RL, Huskamp HA, Mehrotra A. Cannabis April 20th celebration and related emergency department visits. *JAMA Netw Open*. 2025;8(5):e2511635. doi:10.1001/jamanetworkopen.2025.11635

eMethods.

This supplemental material has been provided by the authors to give readers additional information about their work.

© 2025 Lin K et al. JAMA Network Open.

eMethods

Optum Labs Data Warehouse (OLDW) dataset

The Optum Labs Data Warehouse (OLDW) is a longitudinal, real-world data asset with de-identified administrative claims and electronic health record (EHR) data. More details about the dataset can be found in

Optum Labs. *Optum Labs and Optum Labs Data Warehouse (OLDW) Descriptions and Citation.* Eden Prairie, MN: n.p., March 2023. PDF. Reproduced with permission from Optum Labs.

The data has been used in a number of prior publications including:

1. Berlinberg EJ, Kim E, Deiner MS, Patterson C, Porco TC, Acharya NR. Seasonality of herpes zoster and herpes zoster ophthalmicus. *J Clin Virol*. May 2020;126:104306. doi:10.1016/j.jcv.2020.104306
2. Patel SY, Mehrotra A, Huskamp HA, Uscher-Pines L, Ganguli I, Barnett ML. Trends in Outpatient Care Delivery and TelemedicineDuring the COVID-19 Pandemic in the US. *JAMA Intern Med*. Nov 16, 2020 2020;181(3):388-391. doi:10.1001/jamainternmed.2020.5928
3. Steiger K, Herrin J, Swarna KS, Davis EM, McCoy RG. Disparities in Acute and Chronic Complications of Diabetes Along the U.S. Rural-Urban Continuum. *Diabetes Care*. May 1 2024;47(5):818-825. doi:10.2337/dc23-1552

Identifying cannabis related ED visits

ED visits were identified through Current Procedural Technology codes 99281-5. To identify ones that were cannabis-related, International Statistical Classification of Diseases Version 10 diagnosis codes for cannabis related disorders (F12.xx) or cannabis-involved poisoning (T40.7x) in any field.

This strategy is consistent with prior literature Bechard M, Cloutier P, Lima I, et al. Cannabis-related emergency department visits by youths and their outcomes in Ontario: a trend analysis. *CMAJ Open*. Jan-Mar 2022;10(1):E100-E108. doi:10.9778/cmajo.20210142

© 2025 Lin K et al. JAMA Network Open.

Categorizing non-cannabis related diagnoses on ED claims

To better understand what symptoms or diagnoses led to the cannabis ED visit, we identified the first diagnosis code on the claim that was *not* cannabis-related and categorized them accordingly.

- ICD-10 Codes Categorized as Gastrointestinal (e.g. vomiting, nausea):
  A08.xx, E86.xx, E87.xx, K21.xx, K29.xx, K31.xx, K35.xx, K52.xx, K57.xx, K59.xx, K80.xx, K85.xx, K92.xx, O21.xx, R10.xx, R11.xx, R19.xx, S42.xx
- ICD-10 Codes Categorized as Mental Health-related (e.g. psychosis, anxiety):
  F10.xx, F11.xx, F13.xx, F14.xx, F15.xx, F16.xx, F17.xx, F18.xx, F19.xx, F20.xx, F21.xx, F22.xx, F23.xx, F25.xx, F28.xx, F29.xx, F30.xx, F31.xx, F32.xx, F33.xx, F34.xx, F39.xx, F40.xx, F41.xx, F42.xx, F43.xx, F44.xx, F45.xx, F48.xx, F50.xx, F51.xx, F53.xx, F60.xx, F63.xx, F68.xx, F69.xx, F79.xx, F80.xx, F84.xx, F90.xx, F91.xx, F95.xx, F98.xx, F99.xx, G40.xx, G43.xx, R00.xx, R06.xx, R20.xx, R25.xx, R40.xx, R41.xx, R42.xx, R44.xx, R45.xx, R51.xx, R55.xx, R56.xx, S00.xx, S06.xx, T40.xx
- Other ED visits (including visits without an additional diagnosis) categorized as other
- We considered creating a separate category for cyclic vomiting given that the association between cyclic vomiting and cannabis is believed to be driven by chronic cannabis use (vs. a naïve user using excessive cannabis). However, only 2.1% of all cannabis-related ED visits in our sample were associated with cyclic vomiting (ICD-10 diagnosis codes G43.Ax and R11.15) and therefore we included these diagnoses in the gastrointestinal category.

© 2025 Lin K et al. JAMA Network Open.

Subgroup analyses

We conducted subgroup analyses by age group, sex, region of the country, cooccurring diagnoses, state access to open and legally protected dispensaries, day of the week (whether April 20th fell on Friday-Sunday or Monday-Thursday), and time periods (2016-9 vs. 2020-3). We hypothesized there would be a larger risk ratio among young adults due to their greater participation in 420 events, that the risk ratio would be larger when April 20th fell around a weekend facilitating greater participation in 420 events, and that the risk ratio would be larger in 2020-3 given that cannabis potency may have increased in recent years. We also hypothesized that the risk ratio would be larger for those in environments with greater access to open and legally protected dispensaries, and so we calculated it for visits that occurred in state and year combinations in which there was only legal medical dispensaries, legal medical and recreational dispensaries, and no legal dispensaries.

© 2025 Lin K et al. JAMA Network Open.
