# Supplementary material for: Cannabis April 20th Celebration and Related Emergency Department Visits
Source: JAMA Netw Open. 2025 May 21;8(5):e2511635. doi: 10.1001/jamanetworkopen.2025.11635 (PMC12096254; doi:10.1001/jamanetworkopen.2025.11635)
Supplement: Supplement 2. — Data Sharing Statement [file jamanetwopen-e2511635-s002.pdf]

# Data Sharing Statement

Lin. Cannabis April 20th Celebration and Related Emergency Department Visits. *JAMA Netw Open*. Published May 21, 2025. doi:10.1001/jamanetworkopen.2025.11635

**Data**
**Data available:** No

**Additional Information**
**Explanation for why data not available:** We are not allowed to share these data under the DUA we have signed.
